# Bridging the Gap Between Policy and Action: Identifying the Social Security Doctrine in Iran

**DOI:** 10.7759/cureus.88346

**Published:** 2025-07-20

**Authors:** Sayyed Mohammad Moosavy Khatat, Farhad Nosrati Nejad, Mohammad Ali Mohammadi Gharehghani

**Affiliations:** 1 Department of Social Welfare Management, University of Social Welfare and Rehabilitation Sciences, Tehran, IRN; 2 Social Determinants of Health Research Center, University of Social Welfare and Rehabilitation Sciences, Tehran, IRN; 3 Social Welfare Management Research Center, University of Social Welfare and Rehabilitation Sciences, Tehran, IRN

**Keywords:** doctrine, pension, policy implementation, retirement, social security

## Abstract

Introduction

Effective implementation of social security policies remains a key challenge, often due to the lack of coherence between theory, policy formulation, and execution. This study investigates the concept of “doctrine” as a guiding framework for aligning these levels and aims to identify the existing social security doctrine in Iran. In this study, doctrine is defined as a comprehensive, non-prescriptive, and authoritative framework of shared beliefs and principles that pragmatically address policy challenges. It facilitates alignment by offering a shared interpretive lens that enables actors across levels to coordinate actions, reduce ambiguities, and implement policies more consistently.

Methods

This qualitative study followed an integrative design across three sequential phases. First, a systematic literature review was conducted to conceptualize the doctrine and define its characteristics. Second, five experts participated in focus group discussions (FGDs) to identify key challenges and relevant legal sources within the Iranian context. Third, a qualitative content analysis was performed on three core legal documents - the Constitution, the Law on the Structure of the Welfare and Social Security System, and the Social Security Law - along with their legislative deliberations. Data were coded using Atlas.ti version 8.1.3. Each phase informed the next to ensure analytical coherence. Ethical approval was obtained from the University’s Research Ethics Committee, and informed consent was obtained from all participants.

Results

Seven doctrinal domains, comprising 18 categories and approximately 60 codes, were identified: *Missions of the System*, *Basis for Government Intervention in Establishing the Social Security System*, *Support Approach*, *Governance System Interaction with the Social Security System*, *Financing of the Social Security Scheme*, *Covered Population*, and *Conditions for Pension Entitlement*. These domains represent the structural and operational pillars of the Iranian social security system. For instance, the domain “Covered Population” includes categories such as “Vulnerable Groups” and “Employed,” reflecting prioritization in system coverage. The integration of expert insights with legal content enabled the extraction of a grounded and contextually relevant doctrinal framework.

Conclusion

The doctrine-based approach offers a novel mechanism for bridging the gap between policy design and implementation. By fostering shared understanding across policymaking levels, doctrine provides a coherent structure to guide effective policy execution. The methodological framework used in this study is transferable to other social policy domains such as health and education.

## Introduction

We are confronted with the growth of knowledge in the health domain and identification of its determining social, cultural, economic, and environmental components. Consequently, policies and programs in this area find a scope of action at different levels within and outside the health system [1]. On the other hand, policymaking typically takes shape in a political environment, and policymakers, regardless of their intent and knowledge, are influenced by social and economic pressures [2]. Responding to these pressures entails further complexities in policymaking activities. These complexities have posed challenges for researchers and policymakers in establishing effective connections and integrating empirical study results, theories, policies, and implementations. Moreover, these complexities have further impeded policy implementation, especially in cases where policies require a long-term focus, leading to failure [3]. Consequently, there is an increasing need to bridge the gap between scientific theories derived from empirical studies, and policymaking and implementation in the health and social welfare domains.

Parallel to this increase in complexities, the challenges of maintaining coherence across different levels, from knowledge to implementation, have escalated. To overcome this challenge, the research community strives to develop mechanisms, such as knowledge translation, to establish connections between research and its derived benefits [1]. On the other hand, policymakers endeavor to utilize scientific findings in policymaking while monitoring the outcomes of policies at the implementation level [4].

Given the importance of implementing policies in accordance with their goals, implementation knowledge has been studied in various domains over the past decades, including public policy, economics, sociology, public administration, design, medicine, political science, social work, urban planning, education, and public health [5]. A review of the studies conducted reveals that most implementation research primarily aims to achieve a general implementation theory that can provide a comprehensive perspective to explain the observed differences in implementation outcomes while paying less attention to shaping the "implementation" itself [6]. However, today, policy actors and others need proper approaches to translate policy intentions into sustainable and reliable streams of effective public action [6].

In the policy implementation process, reaching an agreement on the nature of the problem and effective interventions can facilitate the implementation of policies and related programs, as designed [5]. However, executives approach toward the logic of change is often based on their beliefs about effective interventions [7]. These beliefs can be considered the product of an interpretive process that is essential for organizational members to understand and share the organization's characteristics, such as its nature, strengths, weaknesses, problems, and solutions [8]. This process forms common interpretive schemas between different levels of decision-making from policymaking to implementation, providing a context for guiding how implementers understand and act on policy guidelines, and directly influencing alignment at policymaking levels. The degree of alignment or divergence between actors' understanding of shared beliefs, particularly regarding what should be done and how it should be done, varies across implementation domains. A strong interpretive culture can provide a set of problems requiring resolution, reduce ambiguity, and, by defining success and failure, evaluate outcomes along their path [9].

The notion of "belief" has previously been employed in the form of the doctrine concept in theology, law, and military sciences. The codification of doctrine in military sciences can be traced back to the fifteenth century [10]. Today, doctrine serves as a strong interpretive culture among the world's armies, particularly the United States Army, which holds a special position. The RAND Corporation's report on reviewing of Army Total Force Policy Implementation identifies doctrine as one of the critical areas of investigation [11].

Because of the nature and role of military affairs in any country, it is highly sensitive, and it is expected that issues from the highest level of command to the lowest level on the frontline are assessed, understood, and resolved with high precision. Therefore, it seems that the use of doctrine, which has evolved in military science over the past five centuries, can significantly assist policymakers and implementers in creating a strong interpretive culture for implementing policies in accordance with the policymaker's intentions.

Despite the application of the doctrine concept in various fields of knowledge, over the past four centuries, it has been increasingly employed in military sciences. Today, the most coherent methods for identifying, codifying, and implementing doctrine have evolved in this domain.

In the realm of implementation, in addition to the quality of the design and program (what), information related to the process (how and by whom) is of great importance. This is something that the military doctrine has addressed with high precision and quality at different doctrinal levels.

By examining the concept of doctrine and doctrinal methodology, this study uses doctrinal frameworks to foster shared understanding and reduce interpretive differences at various levels, including policy formulation, organizational processes, and frontline implementation. This approach can foster interpretive alignment across different levels, enabling programs to be implemented more closely in line with the intentions of policymakers. The specific focus was on identifying doctrine in various domains of social security and retirement funds. While the need for pension reforms is increasingly becoming more pressing, implementing the necessary reforms remains a fundamental challenge [12-14].

Typically, social security in countries employs a pay-as-you-go (PAYG) financing method, which requires implementing reforms in accordance with demographic changes. Failure to do so inevitably leads to a crisis. In democratic systems, these reforms require substantial social support. However, social support is intergenerational because of the nature of the PAYG system, and it is necessary for the current generation to not only bear the burden of the previous generation but also shoulder an additional burden to prevent the situation from worsening for future generations [15].

Preventing crises requires implementing reforms. These reforms are typically based on three pillars: policy design, political implementation, and administrative implementation. However, the importance of implementation is often underestimated, despite policy implementation requiring skills on par with the policy design [16]. Doctrine provides a mechanism to create a shared understanding of goals and methods across different levels, from policy design to managerial implementation.

In response to the persistent challenges in implementing social security policies-and the critical need for alignment across levels of theory, policy formulation, and practice-this study employed a multi-phase, integrative qualitative approach. The research combined three components: a systematic literature review to conceptualize the notion of doctrine; expert focus group discussions (FGDs) to identify fundamental issues and doctrinal sources; and an in-depth qualitative analysis of core legal texts and their legislative deliberations.

Given Iran’s layered legislative history - shaped by multiple foundational laws enacted at different times - and its centralized yet often fragmented implementation system, the country presents a particularly suitable context for applying a doctrinal approach aimed at achieving interpretive coherence. This integrative process not only addresses the inherent complexities in the gap between policy and practice but also fosters shared understanding and reduces interpretive fragmentation among stakeholders, thereby enhancing the alignment between policy intentions and implementation outcomes.

## Materials and methods

Study outline

**Figure 1:**
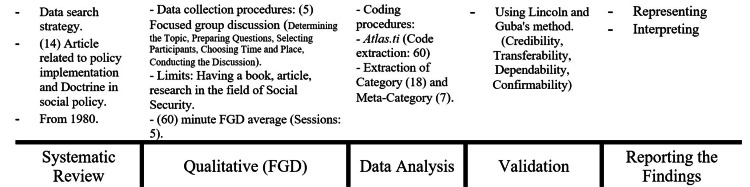
Study outline

This study aimed to identify the social security doctrine in Iran. The outline of this study is presented in Figure 1.

The integration of data across the three phases occurred in a sequential and iterative manner, where the outcome of each phase served as both the input and analytical lens for the next:

Phase One: Literature Review

This phase provided the conceptual foundation of the study. Through a systematic review, five key components of doctrine in the policymaking context were identified. These served as theoretical sensitizing concepts, helping shape the discussions and focus of the next phase.

Phase Two: Focus Group Discussions (FGDs)

The insights from the FGDs were informed by the theoretical understanding developed in Phase One. In these discussions, experts interpreted and contextualized the abstract concept of doctrine in relation to the social security system. Their input led to the formation of 12 initial doctrinal domains, which formed a deductive coding framework for Phase Three. The domains reflected both theoretical relevance (from Phase One) and expert validation (from Phase Two).

Phase Three: Document Analysis

In this phase, the legal and policy documents were coded using the 12 initial domains. However, the analysis evolved inductively, with many new detailed codes (sub-themes) emerging directly from the data. These codes were grouped into 18 categories based on thematic similarity and logical coherence. Eventually, the richness of the data allowed us to refine and reduce the domains to 7 final doctrinal domains. This final structure reflects an integration of** **conceptual grounding (from Phase One), contextual insights and expert framing (from Phase Two), and empirical evidence from documents (in Phase Three).

In short, the data from the three stages were not treated in isolation but were progressively integrated. The literature informed the expert discussions; the expert input shaped the coding structure; and the document analysis enriched, refined, and synthesized the earlier findings. The final doctrinal domains are thus the product of a rigorous and integrated analytical process that combines deductive framing with inductive discovery.

Given the interdisciplinary and complex nature of the subject matter, as outlined earlier, this study employs an integrated approach utilizing a mixed set of qualitative methods that employed in three phases. The first phase included a literature review by conducting a systematic review to identify the meaning of doctrine directed to the field of policymaking. The second phase included FGD to identify the field of welfare and social security issues to use them in the process of qualitative analysis of doctrinal sources, also includes identify doctrinal resources. The third phase includes data collection, coding the data and qualitative analysis of them.

First phase: conducting a systematic review

In the first phase, the concept of doctrine was examined across various texts (political, economic, military, security, and theological) using a search strategy (Table 1) within information databases.

**Table 1:** Search strategy

Database	Strategy
ISI	((TI=(policy OR "policy implementation" OR retirement OR pension OR doctrine OR action OR theory OR practice OR "policy reform")) AND TS=(policy AND pensions)) OR (TI=doctrine AND TS=policy) AND PY=(1980-2024)
Scopus	(TITLE-ABS("policy" OR "policy implementation" OR "retirement" OR "pension" OR "doctrine" OR "action" OR "theory" OR "practice" OR "policy reform") AND INDEXTERMS("policy" AND "pensions")) OR (TITLE("doctrine") AND INDEXTERMS("policy")) AND PUBYEAR AFT 1979 AND PUBYEAR BEF 2025
PubMed	((((policy[Title/Abstract]) OR (policy implementation[Title/Abstract]) OR (retirement[Title/Abstract]) OR (Pension[Title/Abstract]) OR (doctrine[Title/Abstract]) OR (action[Title/Abstract]) OR (theory[Title/Abstract]) OR (practice[Title/Abstract]) OR (policy reform[Title/Abstract])) AND (policy[MeSH Terms] AND pensions[MeSH Terms])) OR ((doctrine[Title]) AND (policy[MeSH Terms]))) AND ("1980/01/01"[Date - Publication] : "2024/11/16"[Date - Publication])

Data Search Strategy

To develop a precise conceptualization of the term “doctrine,” a systematic literature search was conducted across three major databases: ISI Web of Science, Scopus, and PubMed. The search included a broad range of related keywords, such as “policy”, “policy implementation”, “retirement", “pension”, “doctrine”, “action”, “theory”, “practice”, and “policy reform”, applied to titles and abstracts. In addition, subject indexing terms such as policy and pensions (index terms in Scopus and MeSH terms in PubMed) were used to enhance the accuracy and breadth of the search (see Table 1).

In addition to these core databases, supplementary searches were conducted using search engines like Google Scholar to locate additional relevant literature published from 1980 onward, applying similar inclusion criteria. Furthermore, doctrinal and strategic publications from military institutions in the United States, Australia, the United Kingdom, Germany, and New Zealand were also reviewed to understand the methodological roots of doctrinal analysis beyond the social security domain.

**Figure 2:**
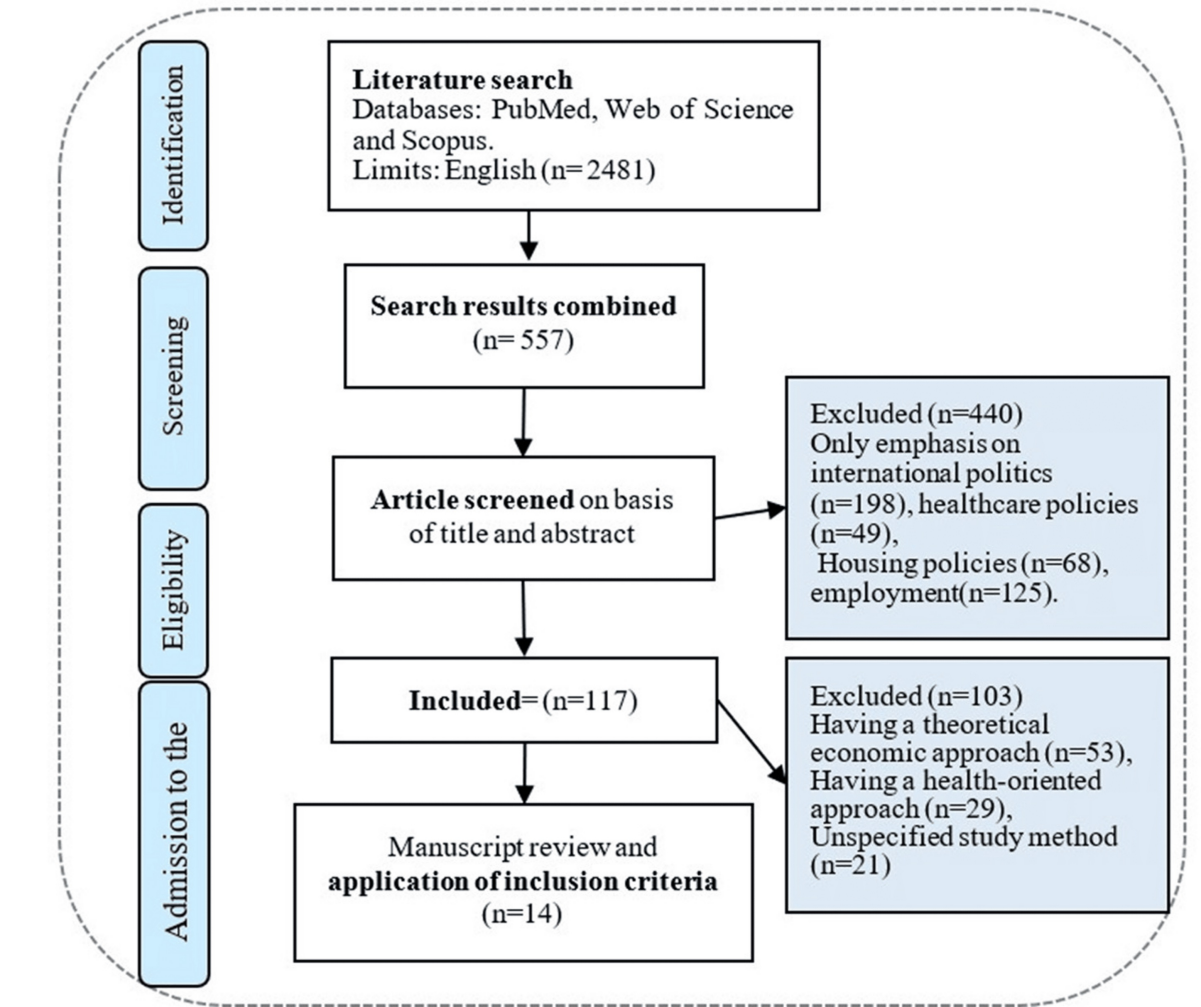
Preferred Reporting Items for Systematic Reviews and Meta-Analyses (PRISMA) flow diagram of the literature search and selection process

A total of 14 articles met the inclusion criteria and were included in this systematic review. The characteristics and main findings of these studies are summarized in the following section. Figure 2 illustrates the rigorous selection process used to identify and screen the relevant studies. This systematic review aims to clarify the multifaceted concept of doctrine within policymaking by extracting key data from these articles, thereby providing a comprehensive understanding of the doctrinal dimensions underpinning social security policies.

Given the conceptual and exploratory nature of the literature review, formal quality appraisal tools such as MMAT or CASP were not applied. However, to reduce the risk of bias and enhance the reliability of the theoretical base, only peer-reviewed journal articles, official institutional publications, and conceptually rigorous sources were included. Sources that lacked transparency or theoretical relevance were excluded.

Second phase: conducting an FGD

Five members participated in the FGD; the main topic was discussing the main aspects of a social security system, for which any designer must have an answer. Another topic was identifying doctrinal sources with the specifications found in the first phase.

The invited people were chosen from those who first had a comprehensive knowledge of the structure of the welfare and social security system, actively participated in the policy-making process, and had at least 10 years of work experience in this field. Prior to participation in the FGDs, all participants received information about the study and signed informed consent forms. Participation was voluntary, and all data were treated confidentially and anonymously. A group interview consisting of five experts was conducted in 60-minute sessions. The selection of this number (five participants) was guided by the specialized and elite nature of the target population, as well as the need to engage individuals with comprehensive knowledge and practical experience at high levels of social security policymaking and legislation. The primary objective was to obtain deep, qualitative insights from distinguished experts, rather than merely collecting data from a large number of participants. This approach prioritizing the quality and depth of participants’ expertise over quantity. The group discussion was moderated in an unbiased manner, ensuring participation and inclusion of all participants' views. To reduce the risk of facilitator bias, the FGDs were led by a trained moderator with prior experience in qualitative research and focus group facilitation. The moderator followed a semi-structured discussion guide, designed in advance based on the study's conceptual framework, to ensure consistency across sessions. Open-ended and neutral questions were used to avoid leading participants or influencing their responses. In addition, the moderator refrained from expressing personal opinions, avoided verbal or non-verbal cues of approval or disapproval, and actively encouraged equal participation by all group members. These measures were aimed at fostering an open and balanced discussion environment.

To ensure the adequacy of data collection, thematic saturation was monitored across the five FGD sessions. After each session, the content was reviewed and compared with previous discussions to identify the emergence of new insights. As the sessions progressed, the responses increasingly converged, with participants reiterating similar viewpoints and no substantially new themes emerging. By the fifth session, it became evident that the discussions had reached a point of saturation, where additional sessions were unlikely to yield novel information relevant to the research objectives. This informed the decision to conclude data collection at this stage.

All FGD sessions were audio-recorded with participants' consent and later transcribed verbatim for analysis. This ensured an accurate representation of the discussions and minimized potential interpretation errors. The transcriptions were reviewed by a second researcher to cross-check fidelity to the original recordings and to strengthen the credibility and trustworthiness of the data.

Third phase: qualitative analysis of doctrinal sources

In this phase, to identify the social security doctrine, the sources of doctrine were first collected based on the findings of the FGD phase; then, they were analyzed via qualitative coding in Atlas.ti version 8.1.3 (Lumivero, LLC, Denver, USA).

The documents that are officially used by the government in the field of social security and pension funds are chosen as doctrinal sources based on the findings of the FGD. These official documents were the Constitution Law, the Law on the Structure of the Welfare and Social Security System, and the Social Security Law, along with the details of their detailed discussions. Because these laws were approved at different times, they included different legislative authorities and legal procedures for approval. These authorities included the National Consultative Assembly, Senate, Assembly for the Final Review of the Constitution, and Islamic Parliament of I. R. Iran. In addition, in some laws, such as the Constitution Law, only some cases were relevant; therefore, the related texts were separated from the details of the deliberations of each of the above assemblies and then consolidated for each law. The list obtained for the Constitution Law included sessions 12-14, 29, 53, 54, and 63 of the Assembly of Constitutional Law Experts, which were held between September 1979 and November 19 of the same year. The details of the negotiations on the Law on the Structure of the Comprehensive Welfare and Social Security System included 13 sessions of the Islamic Parliament of I.R.Iran, which were held over two years from August 2001 to May 2003. The details of the negotiations of the Social Security Law comprised five sessions (four sessions of the National Consultative Assembly and one session of the Senate), which were held from March 1974 to June 1975.

Data analysis

The analysis and interpretation of the documents were conducted using Atlas.ti version 8.1.3, utilizing structural coding (resulting from the second phase of the study). After the initial coding using the main themes of the pension system, similar codes were compared and sub-concepts were identified through secondary coding stage.

In the initial coding, all laws and their detailed discussions were first coded using codes derived from the main topics and concerns obtained from the second phase of the focus group interview. Considering the number of topics used as main codes and the large volume of documents reviewed, the number of texts related to each of the main codes allowed for the creation of subcodes. Therefore, after comparing the quotes related to each code, the subtopics were identified. In addition, some topics did not form good quotations in relevant texts. In addition, under some of the main codes, similar subcodes were formed and merged. Thus, some of the main codes were discarded because of a lack of reliable quotes from the final result. Ultimately, the main domains and their subcodes were identified. Each of these areas, along with their subcodes, forms a doctrinal area, which is presented in the findings section.

Validation

This study was validated within the framework of Lincoln and Guba [17]. In the domain of credibility, expert discussions and member checking were employed in addition to triangulating methods and data. The examination of the study's context across various domains demonstrated its transferability to the realm of social policymaking. The use of Atlas.ti 8.1.3 software for coding enabled the visibility, reviewability, and overall confirmability of all code-related instances and their list. Regarding dependability, the nature of the studied sources, comprising legal documents and their records, was accessible.

Coding reliability

Coding was conducted by the lead researcher. To enhance reliability, the coding process involved iterative reviews, audit trails, and feedback from other members of the research team. In cases of ambiguity or discrepancy, the interpretation of codes was discussed collaboratively within the team until consensus was reached. This approach strengthened the credibility and consistency of the analysis. Additionally, the evolving code structure was continuously compared with the conceptual framework developed in earlier phases to ensure coherence and theoretical alignment.

Ethical considerations

This study received ethical approval from the Research Ethics Committee of the University of Social Welfare and Rehabilitation Sciences (ref. no. IR.USWR.REC.1399.007). Prior to participation, all focus group participants were informed about the objectives, methods, and voluntary nature of the study. Written informed consent was obtained from all participants, and confidentiality and anonymity were assured throughout the research process.

## Results

The majority of articles included in this systematic review, predominantly published between 2022 and 2024, highlight an increasing scholarly emphasis on social security policies and their doctrinal underpinnings. These studies critically explore the interplay between policy formulation and implementation, elucidating evolving doctrinal paradigms within social protection and retirement systems. The thematic breadth, encompassing both theoretical frameworks and empirical analyses, reflects a robust multidisciplinary approach. The integration of contemporary and seminal literature provides a comprehensive understanding of the development and inherent complexities of social security doctrines.

This synthesis is grounded in a rigorous selection protocol, depicted in Figure 2, which outlines the comprehensive methodological framework employed to identify, screen, and select pertinent studies. The characteristics and principal findings of the 14 articles incorporated in this systematic review are succinctly summarized in Table 2.

**Table 2:** Summary of included articles on social security policies and their doctrinal foundations

No.	Article title	Author(s)	Year	Journal	Study type	Geographic focus	Summary of main findings
1	Associations between Retirement, Social Security Policies and Health: A Systematic Review [18]	LM de Oliveira Teixeira et al.	2024	BMC Public Health	Systematic review	Global	Retirement and social security policies have diverse impacts on health
2	Social Protection in the Developing World [19]	Abhijit Banerjee et al.	2024	Journal of Economic Literature	Analytical review	Developing countries	Examines challenges and policy solutions in social protection
3	Pension policies and early retirement: New evidence from a counterfactual analysis in Iran [20]	Saeed Malek Sadati et al.	2024	The Economic and Labour Relations Review	Empirical study	Iran	New evidence on policy effects on early retirement
4	A Comparative Analysis of Current Developments in Studies on the System Secures the Social Sustainability [21]	Yujing Lan, Guan Huang	2024	Pakistan Journal of Life and Social Sciences	Analytical review	Global	Analysis of recent trends in social security studies
5	Retirement’s Impact on Health: What Role Does Social Network Play? [22]	Asal Pilehvari et al.	2023	European Journal of Ageing	Empirical study	Global	Social networks significantly influence retirees’ health
6	Implementing a Public Policy to Extend Social Security to Informal Sector Workers [23]	JJ Miti et al.	2023	Policy Studies Journal	Case study	Developing countries	Challenges and strategies for implementing social security policies
7	Social Protection in Iran: Recent Advances and Challenges Ahead for a More Child-Sensitive System [24]	Mehdi Haji Hosseini et al.	2022	International Policy Centre for Inclusive Growth	Analytical review	Iran	Progress and challenges in Iran’s social protection policies
8	Guiding principles for social security policy: Outcomes from a bottom‐up approach [25]	Michael Orton et al.	2022	Social Policy & Administration	Qualitative study	Global	Presentation of guiding principles developed from participatory methods
9	Does social security policy matter for corporate social responsibility? Evidence from a quasi-natural experiment in China [26]	Wendai Lv et al.	2022	Economic Modelling	Empirical study	China	Social security policies affect corporate social responsibility
10	Ensuring Social Security for All: Key Considerations for Policy Options in Indonesia [27]	Iene Muliati et al.	2021	Policy & Governance Review	Analytical review	Indonesia	Review of policy options and key considerations
11	Policy coherence and social protection in Ethiopia: Ensuring no one is left behind [28]	Melisew Dejene Lemma, and Logan Cochrane	2019	Societies	Analytical review	Ethiopia	Examination of policy coherence to ensure inclusive protection
12	Social Security: A program and policy history [29]	Patricia Martin, and David A. Weaver	2005	Social security Bulletin	Historical review	USA	History and evolution of social security programs
13	Understanding Social Security: Issues for Policy and Practice [30]	S. Wright	2003	Policy Press	Analytical review	Global	Review of policy challenges and practical considerations
14	Extending Social Security: Policies for Developing Countries [31]	van Ginneken et al.	2003	International Labor Review	Policy review	Developing countries	Analysis of policy expansion and related challenges

The results obtained from the first phase, which involved a systematic review of relevant articles and scientific sources, determined the meaning of the doctrine in five components as follows: 1) Resource-grounded: This is derived from a combination of experience, theory, and principles. 2) Pragmatic: Answers practically to defined problems. 3) Optimal: It represents the most effective course of action. 4) Authoritative: To be obedient and acceptable. 5) Non-prescriptive: This is not a prescriptive set of instructions; its application necessitates judgment and contextualization.

To identify the doctrine of social security, in accordance with the second component of doctrine, it must be formulated in response to a practical problem. On this basis, it was necessary to first identify the core issues related to practical problems in the domain of social security, and then identify the responses to these issues. These steps were carried out in the second stage through FGDs. From the notes taken from transcribed content, a list of 12 main issues was extracted, each representing a major concern for designers of the social security and retirement system. These issues and their definitions are presented in the following Table 3.

**Table 3:** List of issues/topics/concerns in the design of social security and retirement systems - derived from focus group discussions (FGDs)

Issue/topic/concern	Definition
Mission of the system	The tasks that must be accomplished to achieve the goals.
Basis for government intervention in establishing the social security system	On what basis has the government intervened to establish the social security system?
Conditions for entitlement to benefits	What criteria are valid for determining eligibility of individuals to receive benefits?
Implementation of the social security scheme	What are the main approaches for selecting the social security scheme for implementation?
Financing	What is the valid financial mechanism for financing the social security scheme?
Covered population	Which segments of the population are prioritized for coverage under the social security system?
Governance interaction with the social security scheme	What are the levels of stewardship and operation of the social security system by the government?
Support approach	On what approaches are the support levels of the social security and retirement system designed?
Targeting mechanism	What is the valid mechanism for identifying target groups?
Provider	How will the provider of social security and retirement services be determined?
Basis for benefit entitlement	What is the role of citizenship rights and financial need in defining benefit entitlement?
Overarching goal	What are the overarching goals of the social security system?

In the third phase, doctrinal sources were selected based on the components identified in the systematic review discussed in the FGD. These official documents containing the doctrine included the Constitution, Law on the Structure of the Comprehensive Welfare and Social Security System, and Social Security Law, along with their detailed discussions. Since these laws were ratified at different times, they involved different legislative bodies and different legal procedures for ratification. These bodies included the National Consultative Assembly, Senate, Assembly of Experts for Constitutional Amendment, and Islamic Consultative Assembly. In addition, in some laws, such as the Constitution, only certain relevant sections were included; therefore, the relevant texts from the detailed discussions of each of the aforementioned assemblies were separated and consolidated for each law. The list obtained for the Constitution included sessions 12-14, 29, 53, 54, and 63 of the Assembly of Experts for Constitutional Amendment, which were held between September and November 1979. The detailed discussions of the Law on the Structure of the Comprehensive Welfare and Social Security System comprised 13 sessions of the Islamic Consultative Assembly held over approximately two years from July 2002 to April 2004. Detailed discussions of the Social Security Law consisted of five sessions (four sessions of the National Consultative Assembly and one session of the Senate) held from March to June 1975.

After gathering data, the analysis and interpretation of the documents were conducted using Atlas.ti version 8.1.3 software, employing structural coding (Table 3) derived from the second phase of the study (FGD). Following the initial coding based on the primary issues of the pension system, similar codes were compared, leading to the identification of sub-concepts through sub-coding. During the initial coding process, all the laws and detailed discussions were coded. Given the number of issues used as the main codes (Table 3) and the large volume of documents under review, the number of texts related to each of the main codes was such that it allowed for the creation of subcodes. Therefore, after comparing the relevant quotes for each code, the subcodes were identified. Additionally, some issues did not generate good quotes in relevant texts. Furthermore, similar subcodes emerged under some of the main codes, which were then merged. Thus, some of the main codes were excluded from the final result because of a lack of reliable quotes. Finally, a list of 60 codes was created during the second round of coding (Table 4).

**Table 4:** List of codes derived from the study

Derived codes				
Retirement insurance	Unemployment insurance	Accident insurance	Disability insurance	Health insurance
Indexation of pensions to inflation	Tax-exempt benefits	Appropriate replacement rate	Compulsory insurance	Compulsory insurance premium
Expansion of compulsory insurance	National cohesion	Meeting basic human needs	Mitigating unjust impacts of decisions	Promoting welfare
Improving quality of life for all	Reducing population below poverty line	Subsidies for low-income households	Combating exploitation	Job creation
Necessity of livelihood provision	Determining minimum benefits	Healthcare should not be income-dependent	Benefit payments based on insurance premiums from continuous benefits	Benefit payments based on
Benefit payments based on wages from multiple employers Share Rewrite	Pension payments based on estimated income	Policymaking	Planning	Resource provision
Supervision	Reducing operational role	Independence of funds	Operating the scheme as an enterprise	Revenues from fines and damages
Tax Exemptions and Judicial Costs of the Organization	Revenues from public property	Other revenues from religious rulings	Supplementary insurance	Payroll deductions
Profits from Securities	Proceeds from business operations	Proceeds from bank deposits	Vulnerable dependents	Victims of accidents
Rural residents	Women	Elderly	Children	Temporarily unemployed
Self-employed and freelancers	Government employees	Armed forces personnel	Covered by labor law	Employers
Official retirement age	Early retirement	Minimum service period	Standard service period	Maximum service period
Total disability	Death	Unemployment		

In the next step, the codes were organized into 18 categories in seven domains. Each of these seven domains, along with their categories and sub-codes, forms a doctrinal domain, which is presented in Table 5.

**Table 5:** Iranian Social Security Doctrine Derived from the Study of the Constitution, the Law on the Structure of the Comprehensive Welfare and Social Security System, and the Social Security Law

Category	Issues/domain	Doctrine
Insurance coverage	Missions of the system	Social Security Doctrine
Pension adequacy		
Mandatory savings		
Demand for social cohesion	Basis for government intervention in establishing the Social Security System	
Demand for welfare programs		
Demand for economic justice		
Maintaining minimum livelihood (relative poverty)	Support approach	
Income maintenance		
Stewardship	Governance system interaction with the Social Security System	
Operational role		
Public resources	Financing of the Social Security Scheme	
Insurance premiums		
Investment		
Vulnerable groups	Covered population	
Employed		
Official retirement age	Conditions for pension entitlement	
Service period		
Occurrence of contingencies		

## Discussion

This study aimed to introduce doctrine as a tool to align theory, policy, and implementation in the realm of policymaking and fill the gap between them; in this regard, we needed to have a practical concept of doctrine for use in policymaking. After conceptualizing the meaning of the doctrine used in policymaking, a method is required to derive an existing doctrine in a particular policy area. Therefore, a method has been developed to identify the existing doctrine of social security in Iran. Ultimately, the final result of this study is the doctrine of social security in Iran. To this end, efforts were made in three stages: Initially, the concept of doctrine was extracted through a literature review, resulting in the identification of five components (resource-grounded, pragmatic, optimal, authoritative, and non-prescriptive) for doctrine. In the next stage, based on these components, a framework containing the social security doctrine was developed through focus group discussion. As a result of the group discussion and analysis, 12 main domains emerged and doctrinal resources were also found. In the third stage, doctrinal sources, including three fundamental laws - Constitution Law, the Law on the Structure of the Welfare and Social Security System, and the Social Security Law - along with detailed discussions of these laws in the relevant decision-making bodies, were gathered, analyzed, and interpreted through coding. Finally, the doctrine of social security in Iran was identified, which included seven main domains and 18 categories.

In examining the challenges facing the implementation of social security and retirement policies in Iran, Vesali et al. [32] identified weaknesses in the implementation of laws in this area, and Omidi [33] attributed a significant portion of this weakness to contradictions at different levels of law formulation, which ultimately manifested itself in the form of contradictory interpretations of the legal text. Creating harmony and integration at different levels of formulation and implementation is a capacity that doctrine possesses and can combine at different levels from theory to practice [34].

In this regard, the present study examines the concept of doctrine for application in the field of social policymaking. The concept inferred in this study is consistent with that of doctrine in various sources and studies [35,36].

There are different views on whether a doctrine should be descriptive or prescriptive. However, at the very least, doctrine is expected to provide a coherent framework that integrates concepts, foundations, and principles internally and guides the design and conduct of operations [37]. Therefore, in this study, efforts were made to ensure that the identified doctrine could provide a coherent framework for integrating principles, foundations, and concepts to guide social security policymakers and implementers.

While the primary aim of this study was to identify the existing social security doctrine in Iran, it also contributes to understanding how doctrine formulation patterns may guide future efforts to systematize and operationalize policy frameworks in social security. While avoiding engaging in the doctrine formulation process, it has pursued the identification of doctrine with a quality that can meet at least the minimum expectations of doctrine. To this end, it has employed the patterns and methods of doctrine formulation as a guide. In doctrinal studies, a wide range of patterns for doctrine formulation has been introduced. This range extends from three stages [38] to eight stages [39]. The model presented by Danesh Ashtiani [38] includes the stages of information gathering, doctrine formulation and development, and the publication stage. On the other hand, the model presented by Drew [39] includes experience/ theory/ technology, integration and analysis, concept development, testing/ evaluation/ discussion/ debate/ argument, acceptance/ rejection/ combination, writing and publication, force training, and application, comprising eight stages. The present study does not include the publication stage in Danesh Ashtiani's model and the publication, force training, and application stages in Drew's [39] model. This study confirms the intermediate five-stage model developed by [40], which includes the five stages of information gathering, planning, production, implementation and operationalization, and feedback.

In addition to patterns, numerous methods have also been introduced for doctrine formulation. The four major types include bottom-up [41], top-down [42], transformation of individual doctrine to organizational doctrine, and experience-based [38]. Considering the processes employed, the underlying method used in this study was the bottom-up approach. However, the primary objective of the studied laws was not doctrine formulation, and in reviewing the detailed discussions, the representatives also expressed their views during the sessions based on the subject matter and within the framework of the proposed articles. Therefore, the overall shaping and structure of the doctrine were carried out through the application of the top-down method, which was presented as the second stage in the review method. The combination of these two methods helps to ensure that both the statements and the structure are based on empirical documents, thereby doubly increasing the validity of the work.

In the policy implementation literature, two important analytical frameworks can be distinguished, bearing similar names to the two doctrine formulation methods: the bottom-up approach and the top-down approach [43]. The top-down approach starts from a sovereign decision, such as a law, and focuses on which objectives have been achieved over time and why this achievement has occurred [44]. The bottom-up approach begins by identifying a network of individuals involved in service delivery in one or more areas and questions these individuals about their goals, strategies, activities, and relationships. It then utilizes these relationships to identify higher levels of decision-making and policymaking, up to the highest level. The bottom-up approach provides a mechanism for moving from the lowest level of implementation to the highest level of policymaking in both the private and public sectors [45].

The present study, insofar as it focuses on the analysis of laws, can be placed within the top-down analytical framework. However, since it has examined the initiation of discussions and the views of individuals involved in the enactment of the law, it can be associated with the bottom-up analysis framework. Ambiguity in the bottom-up approach is greater than in the top-down approach [46], and on the other hand, a fundamental characteristic of doctrine is to guide actors at different levels. Therefore, it is expected that doctrine will benefit from the bottom-up approach for greater comprehensiveness, but ultimately, it must be formulated in a way that reduces ambiguity at different levels.

To better explain the issue, Matland's [46] ambiguity-conflict model can be utilized. On the one hand, doctrine is potentially exposed to high conflict from actors due to its application in different environments and at different implementation levels, and on the other hand, doctrine can increase the level of conflict by reducing ambiguity. At the same time, however, doctrine particularly increases convergence in objectives, which can ultimately transform doctrine into an effective tool for the precise implementation of the policymaker's intentions. Matland's [46] model shows that by moving from the symbolic situation, where both conflict and ambiguity levels are high, to the administrative situation, where goals are agreed upon, the objective utility function can be maximized [47].

The identified social security doctrine has several limitations. One limitation of this study was the small sample size (n = 5) in the FGD, which may affect the comprehensiveness of the extracted doctrine. However, this number was selected considering the specialized nature and limited access to expert participants, as explained in the Methods section, leading to data saturation in in-depth discussions. In addition, the doctrine is limited to the main laws and does not encompass secondary laws, regulations, or bylaws. Moreover, the scope of the doctrine depends heavily on the interpretation of the concept of social security, which can be understood broadly from a social perspective [48] or narrowly, focusing solely on retirement.

Furthermore, the doctrine has the potential to harmonize theoretical knowledge with approved policies and their implementation. For instance, some studies have highlighted psychological risks associated with retirement [49,50]; however, retirement policies are predominantly influenced by financial and economic considerations. In this context, the doctrine could provide a path forward, but this aspect was not explored in the current study. Nevertheless, the findings of this study provide grounds for learning new methods and tools for applying doctrine in the field of social security policymaking and policy implementation. The conscious formulation of the social security doctrine based on relevant patterns and methods can be considered in future research. Aligning doctrinal methods with empirical and qualitative methods was one of the main challenges of the present study. The method chosen in this study was based on the necessity of the study's objective, which was to identify the social security doctrine, and in the event of a change in the objective toward doctrine formulation, it can be modified according to the requirements of each stage. Acknowledging these limitations, this study has provided a method through which the general frameworks of the doctrine governing the studied area can be clearly obtained, and these findings are supported by the data.

## Conclusions

This study provides new empirical insight into the persistent challenges of policy implementation in Iran’s social security system by introducing a structured and context-specific doctrinal framework. Rather than reiterating the conceptual gap between policy design and execution, the research identifies seven doctrinal domains-comprising 18 categories and approximately 60 codes-derived from analysis of legal documents and expert perspectives. These domains represent an interpretive structure that clarifies how key issues such as coverage, financing, entitlements, and governance are formally addressed within the system. By making these structures explicit, the doctrinal framework serves as both a diagnostic lens and a practical guide to enhance coherence between different levels of policymaking and implementation.

Iran’s layered legislative history and centralized yet fragmented implementation structure make it a particularly suitable context for applying the doctrinal approach. These findings are therefore especially relevant for policymakers seeking alignment between institutional rules and policy goals in complex governance environments.

While the present study focused on the social security domain, the structured doctrinal approach developed here may serve as a foundation for future research into its applicability across other areas of social policymaking, such as health or education. Moreover, the doctrinal framework may also be relevant to other countries that share similar structural and institutional features with Iran.
